# Use of RNA Interference by *In Utero* Electroporation to Study Cortical Development: The Example of the *Doublecortin* Superfamily

**DOI:** 10.3390/genes3040759

**Published:** 2012-11-21

**Authors:** Orly Reiner, Anna Gorelik, Raanan Greenman

**Affiliations:** Department of Molecular Genetics, Weizmann Institute of Science, 76100 Rehovot, Israel; E-Mails: anna.gorelik@weizmann.ac.il (A.G.); raanan.greenman@weizmann.ac.il (R.G.)

**Keywords:** doublecortin, DCX, shRNA, *in utero* electroporation, neuronal migration

## Abstract

The way we study cortical development has undergone a revolution in the last few years following the ability to use shRNA in the developing brain of the rodent embryo. The first gene to be knocked-down in the developing brain was *doublecortin (Dcx).* Here we will review knockdown experiments in the developing brain and compare them with knockout experiments, thus highlighting the advantages and disadvantages using the different systems. Our review will focus on experiments relating to the *doublecortin * superfamily of proteins.

## 1. Introduction

The human cerebral cortex is the seat of cognition and as such, a focus of many intensive studies. In humans, brain malfunctioning may be detected early in life since it will impact normal development. During early childhood, children are carefully monitored to reach developmental milestones, which include motor functions, cognitive abilities, development of language capabilities and social interactions. Therefore, it is not surprising that many genes associated with brain diseases were first identified in humans. Nevertheless, the human brain is not readily accessible and studies that are aimed at understanding the developmental and cellular bases of such diseases rely on model organisms. In many aspects the mouse brain is similar to the human brain, and therefore it is a commonly used model. The mouse develops a six-layer cerebral cortex, similar to humans, yet the time frame of mouse brain development occurs within days, *versus* months in the human brain. Most importantly, the mouse embryonic brain is accessible for *in vivo* manipulations*.* In addition, naturally existing and engineered mouse mutants provide endless options for investigating processes of brain development.

The most common approach to gene function study involves gene knockout or knock-in; this approach is expensive and time-consuming. One of the relatively recent additions to the wide repertoire of nucleic acid molecules used to silence gene expression are small interfering RNAs (siRNAs) [1]. siRNAs were first discovered in the nematode *C. elegans*, where the injection of double-stranded RNA initiated sequence-specific degradation of cytoplasmic mRNA [2,3]. In the developing brain, the vector based short hairpin RNA (shRNA) is more commonly used than the chemically synthesized double-stranded siRNA. The introduction of shRNA in the developing brain may overcome an important disadvantage in regard to gene knockout; sometimes the expected phenotype is not observed in the respective mouse model. It has been speculated that this may be due to genetic compensation and/or species differences. This hypothesis was first raised when shRNA of *doublecortin (Dcx)* was introduced into the rat developing brain [4]. Here, we will review the usage of shRNA to study cortical development using the *doublecortin* superfamily as an example.

## 2. Introduction of shRNA in the Developing Brain: Pros and Cons

### 2.1. Silencing of Gene Expression

RNA interference (RNAi) is the process by which dsRNA silences gene expression, either by inducing the sequence-specific degradation of complementary mRNA or by inhibiting translation [5]. In a wide range of organisms, double-stranded RNA triggers posttranscriptional gene silencing or RNA interference (RNAi). Genetic and biochemical investigations of the mechanisms guiding RNAi in different organisms revealed the conservation of cellular machinery that cleaves long dsRNA into duplexes of 21- to 28-nucleotide siRNAs, which guide the sequence-specific degradation of mRNAs. siRNA, shRNA and miRNA elicit RNAi through common biochemical pathways involving complexes of enzymes including Dicer [6,7,8,9]. The siRNAs are then incorporated into the RNA-induced silencing complex (RISC), which unwinds the duplex siRNA into single-stranded siRNA [10]. The antisense strand of the duplex siRNA guides the RISC to the homologous mRNA, where the RISC-associated endoribonuclease cleaves the target mRNA at a single site in the center, which results in the silencing of the target gene [6,11]. One caveat of siRNA design is that not all 19–22 base RNA duplexes will cleave their target with efficacy. A key finding, which improved the design was when it was found that the RISC complex is asymmetric and favors the strand of the siRNA duplex with the least thermodynamically stable 5' terminus [12,13]. This information was used in the design of several algorithms to better select an effective siRNA target site within a gene [14,15]. Small interfering RNA (siRNA) and short hairpin RNA (shRNA) have been initially proven effective in reducing gene expression in cultured mammalian cells [6,16,17]. Later, gene silencing has been shown to be effective in rodents and is being considered for a therapeutic strategy in humans (reviews [5,18]).

### 2.2. Gene Redundancy

In mammals, *DCX* is part of a small superfamily of proteins, defined by the presence of a conserved microtubule-binding domain, the DCX domain [19,20]. Mutations in the founding member of this gene family were found to cause X-linked lissencephaly (“smooth brain”) in males and double cortex syndrome in females [21,22]. Further studies revealed that additional members of this gene family are involved in an array of neurological disorders. Its closest family member is *Doublecortin like kinase-1*, *DCLK1*, which has recently been implicated in cognitive function in humans [23]. Rare copy number variations of *Doublecortin like kinase-2*, *DCLK2*, were detected in a study investigating attention deficit hyperactivity disorder (ADHD) [24]. *Doublecortin domain containing 2*, *DCDC2*, has been suggested to play a causative role in reading disabilities and has been implicated as a susceptibility gene for Dyslexia [25,26]. *Retinitis Pigmentosa 1*, *RP1*, and *Retinitis Pigmentosa like 1*, *RP1L1*, are associated with inherited blindness [27,28]. The known CNS expression pattern and possible function of the DCX gene superfamily are summarized in Table 1.

**Table 1:** The doublecortin superfamily of proteins, expression in the rodent CNS, and putative functions there.

Protein	Expression	Function
**DCX**	The developing neocortex [21,29], olfactory cells, developing retina [20], adult neurogenic regions [30,31]	Neuronal migration during development [4,32,33]; migration of adult SVZ cells [34]; branching of neurites, dendrites [32,35,36]; epilepsy [17]
**DCLK1**	Developing neocortex [20,37,38], olfactory cells [20], adult brain [31,39], developing retina [20], neuronal progenitors [31]	Neuronal migration during development [32,39]; neurogenesis [38]; apoptosis [40,41]; hippocampal activity, anxious behavior, contextual fear memories [42,43,44]
**DCLK2**	Developing neocortex [45,46], adult brain [45], developing retina [20]	Hippocampal lamination [47]; branching of dendrites (hippocampus) [47]
**DCLK3**	Adult brain [48]	
**RP1**	Retina, part of the photoreceptor axoneme [49]	Microtubule organization [49], organization of the photoreceptor outer segment [50]
**RP1L1**	Developing retina [20,28]	
**DCDC2**	Cortex [26], choroid plexus, cerebellum [20]	Neuronal migration [26], structure and function of primary cilia [51]
**DCDC2B**	Developing neocortex [20]	
**DCDC2C**	Ubiquitous expression [20]	
**DCDC5**	Thalamus, posterior hypothalamus, septum, developing retina, olfactory cells, choroid plexus [20]	Mitosis [52]

Despite the severe brain malformation observed in male patients carrying mutations in the *DCX* gene, the mouse knockout did not exhibit the expected inhibition in the migration of pyramidal neurons in the developing brain [53]. However, knockdown of *Dcx* using *in utero* electroporation inhibited migration of cortical excitatory neurons both in rat and in mouse brains [4,54]. It has then been suggested that the lack of the radial migration phenotype in the knockout models may be due to gene redundancy. It is postulated that whereas in case of the knockout there is plenty of time for developmental redundancy mechanisms to become operative; the knockdown involves an acute gene reduction, which may not allow sufficient time for redundancy mechanisms to evolve. This notion received additional support following findings that the knockout of *Dclk1* did not result in an observable phenotype in the migration of pyramidal neurons in the developing brain [39,55]. Similar to *Dcx*, the knockdown of *Dclk1* impaired the migration of pyramidal neurons [55]. Nevertheless, the double knockout of *Dcx* and *Dclk1* had a clear effect on cortical development. More specifically, the double mutant mice demonstrated perinatal lethality, disorganized neocortical layering and profound cytoarchitectural defects of the hippocampus caused by the disruption of radial neuronal migration. In the adult *Dcx* mutant mice some deficits were noted in the migration of neurons to the olfactory bulb in the rostral migratory stream [56]. The possibility of gene redundancy was investigated in *Dcx* mutant mice, where the expression of transcripts and proteins, which are products of the *Dclk1* and *Dclk2* gene, were analyzed [46]. A minor change in the expression of one of the DCLK1 proteins was detected in this study. In addition, more severe phenotypes were noted in the combination of mutant alleles for *Dcx* and *Dclk2* [47]. In particular, in the absence of *Dcx* and *Dclk2* there was a dosage-dependent phenotype in the hippocampus, where hippocampal lamination was disrupted and it was accompanied with simplification of pyramidal dendritic arborizations. However, as mentioned above, the DCX protein family includes additional family members, which may participate in gene redundancy mechanisms. *Dcdc2*, an additional family member, was suggested to exhibit functional redundancy with DCX. Knockdown of *Dcdc2* inhibited neuronal migration, whereas *Dcdc2^−/−^* mice revealed no obvious phenotypes in neuronal migration, neocortical lamination, neuronal cilliogenesis or dendritic differentiation [26,57]. The investigators tested whether decreasing *Dcx* expression by RNAi would differentially impair cortical development in *Dcdc2* knockouts and wild-type mice. Consistent with this hypothesis, they found that deficits in neuronal migration, and dendritic growth caused by RNAi of *Dcx* were more severe in *Dcdc2* knockouts than in wild-type mice. These results suggest that *Dcdc2* exhibits partial functional redundancy with *Dcx*. These conclusions could not have been reached relying on mouse knockout data exclusively. Thus, the implementation of knockdown technology in the developing brain and the combination of knockout and knockdown approaches allowed for better understanding of gene function.

### 2.3. Controls, Off-Targets

Efficacy and specificity are two important issues that need to be addressed in a siRNA/shRNA system. Despite the fact that very sophisticated algorithms are being used to design the sequences, experimentally, only a portion of the sequences used in shRNA constructs is efficient in reducing the desired mRNA. Part of the problem may be due to the relative inaccessibility of the short sequence to form a duplex with the mRNA, due to the complex three-dimensional structure of the mRNA. This shortcoming may yield false negative results; however, usually it is possible to verify whether a gene has been successfully knocked down. Common assays include assaying the levels of the mRNA using real-time PCR (qPCR) of the target gene or *in situ* hybridization. If antibodies are available, then it is possible to test the reduction in the expression of the target protein by western blot analysis and/or immunostaining. However, it needs to be considered as a drawback when gene knockdown verification is not practiced, for example, in many high-throughput screens. 

Target specificity is an additional critical step required for successful gene silencing. Off-target effects are driven by three main mechanisms: activation of the nonspecific immune response (such as the interferon response), saturating cell machinery and reduction of non-targeted mRNA (reviews [58,59]). Several practical steps are used to minimize unintended effects. Most design programs examine the existence of identical or near identical sequences in the genome using BLAST-based analysis. However, expression-profiling studies have demonstrated that a small degree of similarity is sufficient to result in off-target gene regulation [60]. In some cases, the off-target event may be due to converting a siRNA sequence into a miRNA (micro RNA), which requires less bases of exact homology for inhibition of translation through a pathway closely related to siRNA [59,61]. Therefore, siRNA sequences that are short and contain perfect matches of 11 bp to other genes are usually to be avoided. In each experiment, several sequences are tested for their specificity and efficacy. Usually, several concentrations of the siRNA are tested and experiments should be conducted with the lowest effective concentration. Sometimes, a combination of several sequences targeting a common mRNA is used to increase efficacy. Negative controls, which include either sequences with multiple mismatches, or sequences with no known homologies, or sequences with confirmed minimal effect assayed by high throughput approaches are used. Other types of controls include occasionally scrambled shRNAs or mismatch shRNA sequences. These controls are not always recommended, since in both cases it is possible that these controls may affect the expression of the target endogenous gene or off-target genes via microRNA activity, which require much less sequence identity, as described above. Finally, the ultimate control is a rescue experiment, which will be discussed below. A summary of the items discussed above is found in Table 2.

**Table 2:** Usage of knockdown *in utero* experiments to study the developing brain: pros and cons.

PROS	CONS
▪ Shorter time frame than knockout ▪ Allows to overcome compensatory mechanisms of gene redundancy ▪ Combinatorial knockdown of two or more genes may be easily performed ▪ Different brain regions can be targeted ▪ Time window of targeting is from E10 to adult ▪ Both cell-autonomous and non cell-autonomous aspects can be studied	▪ Proper controls are needed to rule out off-target and non-specific effects ▪ Special equipment is required ▪ Difficult for early developmental processes

### 2.4. Rescue

The rescue experiment involves expression of a construct of the relevant cDNA of the knocked down gene, which is resistant to the shRNA. The cDNA is introduced with the shRNA, and in case of a successful rescue, the functional phenotype is ameliorated. Resistance of cDNA to the shRNA can be achieved either when the complementary sequence is absent (for instance when it resides in the UTR), or when it contains silent mutations corresponding to the shRNA sequence. This is an excellent control, yet in some cases it may be very difficult to attain. Many of the genes involved in regulation of neuronal migration are dosage sensitive, thus reduced expression or elevated expression, which might be caused by the expression of the resistant mRNA, results in an apparent phenotype. For example, overexpression or reduced expression of the microtubule regulating kinase, MARK2/Par-1, affected neuronal migration [62]. A rescue experiment was successful only after tittering down the expression levels and controlling the temporal expression pattern using different promoters [62]. In the case of the DCX protein family, both gain and loss of function of Dclk1 disrupt the cell cycle progression of neuronal precursors, suggesting that very precise levels of Dclk1 are required for completion of mitosis [38]. Both knockdown and overexpression of Dcx impair neuronal migration [63]. Thus, very subtle changes in the concentration of DCX are required to achieve rescue. Rescue experiments can provide more information than just confirmation of specificity. The re-expression of Dcx at the postnatal stage restored developmental defects caused by the introduction of Dcx shRNA in the developing brain of the embryo [64]. Thus, this study demonstrated the existence of a time window in which the neurodevelopmental deficiency could have been reversed (review [65]). In addition, rescue experiments using other genes are very powerful to demonstrate genetic interactions. For example, DCX is phosphorylated by MARK2/Par-1, which resulted in its reduced affinity to microtubules [66]. We have shown that the reduction in DCX destabilized microtubules, while reduction in the cellular levels of MARK2 stabilized microtubules by alleviating its effect on DCX [67]. Subsequently, co-reduction of both proteins resulted in partial restoration of a normal migration pattern *in vivo*, and in partial restoration of centrosomal motility [67]. Thus, combinatorial additions of different shRNA may provide useful insights regarding genetic interactions.

### 2.5. Targeting Different Areas or Different Cell Populations

The cerebral cortex is an intricate structure composed of different areas, layers and cell types; each of which originates at a distinct place and unique time during development. Studying the development of individual cell populations requires the ability to manipulate discrete groups of cells at different times. The profuse variety of knockdown methods allows for a refined regulation over the cortical population or the area that is being knocked-down. Regulation, both temporal and spatial, is possible by the use of conditional RNAi-constructs (*i.e.*, Cre mediated conditional RNAi activation or inactivation and drug-inducible RNAi) and by the RNAi-construct transfection system.

**Figure 1:**
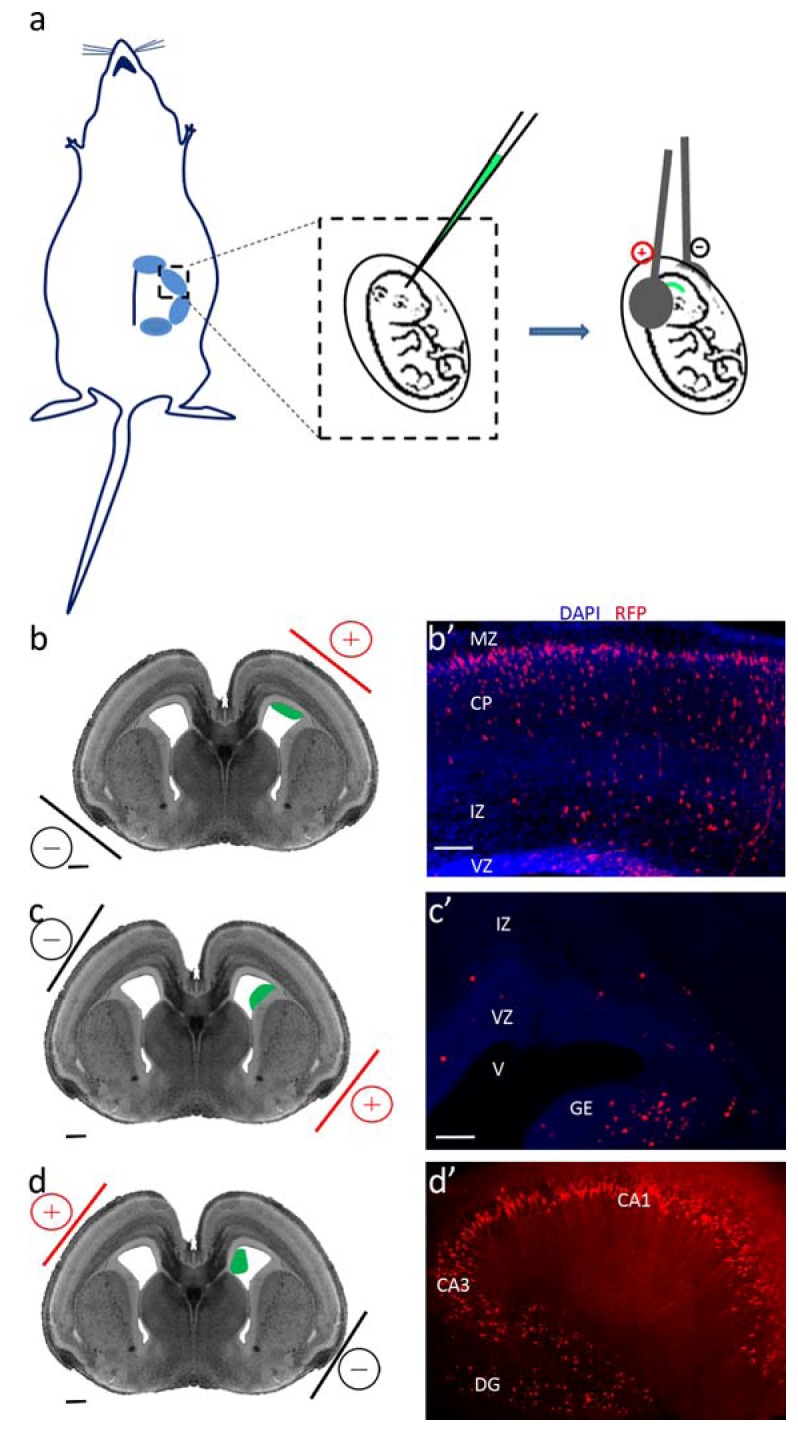
*In utero* electroporation enables spatial regulation of shRNA transfection. (**a**) Schematic representation of the *in utero* electroporation technique. (**b**–**d**) The position of the positive pole of the electrode determines the cortical areas to be targeted. Targeting the ventricular zone of the dorsal telencephalon (b) labels cortical projection neurons (b’ our unpublished data). Targeting the ganglionic eminences of the ventral telencephalon (c) labels cortical interneurons (c’ our unpublished data). Targeting the hippocampal neuroepithelium (d) labels hippocampal neurons (d’); image adopted with permission from [71]). Embryos were electroporated *in utero* at E14.5 (b–d; scale bar: 250 µm) and harvested at E18.5 (b’–c’; scale bar: 100 µm) or P15. DAPI, 4',6-Diamidino-2-phenylindole dihydrochloride; RFP, red fluorescent protein; CP, cortical plate; DG, dentate gyrus; GE, ganglionic eminences; IZ, intermediate zone; MZ, marginal zone; V, ventricle; VZ, ventricular zone.

Currently, two systems for transfection of RNAi-constructs into the rodent brain are utilized: viral transduction (for example [38]) and *in utero* electroporation [68,69], where the latter is more commonly used. It should be noted that in both methods involve injection into the brain, thus involving extremely skillful experimentation of well-trained persons. Mechanical damage of the tissue may evoke the response of the immune system. In case of *in utero* electroporation, the uterine horns are exposed and shRNA constructs, usually together with a fluorescent-protein, are microinjected into the lateral ventricle of the embryo’s brain (Figure 1a). The DNA is incorporated into the cells at the vicinity of the ventricular surface by the application of a series of square-pulse waves. Depending on the position of the positive pole of the electrode, different cortical areas can be targeted. By targeting the ventricular zone of the dorsal telencephalon, populations of the cortical projection neurons can be affected (Figure 1b) [68,69]. Most of the abovementioned experiments, targeted at investigating radial migration in the developing cortex, used this configuration [4,54,62,67]. However, slight changes in the position of the electrodes can result in labeling of other cell populations. For example, successful transfection of neurons in the lateral cortical stream in the rat brain was achieved [33]. These cells contribute neurons to structures in the ventral telencephalon including the amygdala and piriform cortex. The authors noted that they were able to transfect cells at the corticostriatal junction without significant contamination of cells in the ganglionic eminence despite the proximity of these structures. RNAi of either *Dcx* or *Lis1* significantly affected migration of neurons to these structures, yet the knockdown of the two different genes did not result in the exact same phenotype. Two additional populations of cortical neurons that can be manipulated are the GABAergic interneurons, which originate in the ganglionic eminences of the ventral telencephalon (Figure 1c), and the hippocampal neurons, which originate at the hippocampal neuroepithelium (Figure 1d) [70,71]. Furthermore, small-diameter electrodes enable expression in a rather small, localized area [65]. Projection neurons compose the majority of telencephalon neurons; thus, most of the research employing *in utero* electroporation is aimed at this population. Nonetheless, knockdown of *Dcx* and *Dclk1* in the ganglionic eminences showed inhibited migration of cortical interneurons [32]. Moreover, the knockdown of *Dcx* in interneurons, but not the knockdown of *Dclk1*, increased branching, suggesting that DCX and DCLK1 act through different mechanisms.

Cortical neurons are organized in layers according to their birthdates [72], therefore, choice of the timing of electroporation can label different cortical layers [73]. Thus, manipulating both the position and timing of the *in utero* electroporation can target distinct cortical populations.

**Figure 2:**
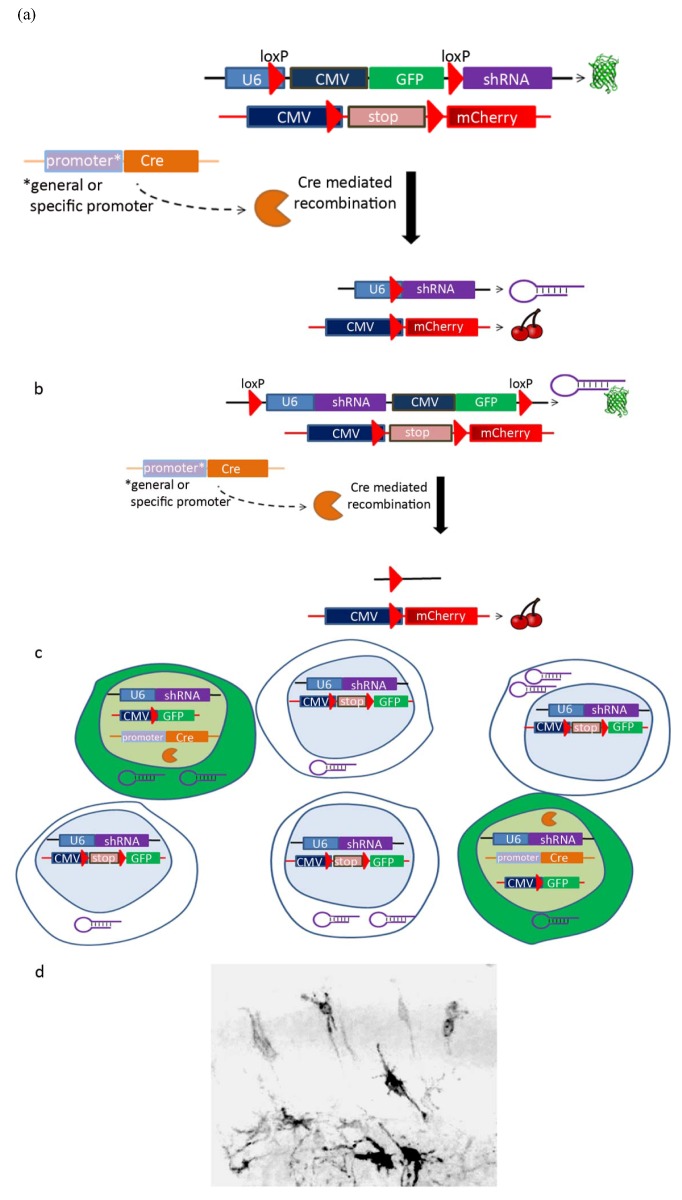
Design and mode of action for conditional knockdown approaches mediated by Cre recombinase. (**a**) Example of a strategy for Cre-mediated shRNA activation. Three DNA constructs are electroporated into the brain: a Cre recombinase under the control of a general or cell-specific promoter, an shRNA containing a DNA stuffer sequence flanked by loxP sites between a U6 pol III promoter and the shRNA coding sequence, and a fluorescent protein (mCherry) containing a stop cassette flanked by loxP sites between a general promoter (CMV) and the mCherry coding sequence. The stuffer sequence of the shRNA plasmid contains GFP under the control of a general promoter (CMV), therefore without Cre activity GFP is expressed. Upon Cre-mediated recombination, the stuffer sequence and the stop cassette are excised and thereafter the shRNA and mCherry fluorescent protein are expressed. (**b**) Example of a strategy for Cre-mediated shRNA inactivation. As in (a), three DNA constructs are electroporated: a Cre recombinase under the control of a general or cell-specific promoter, an shRNA that also contains a GFP marker such that both shRNA and GFP are flanked by loxP sites, and a fluorescent protein (mCherry) containing a stop cassette flanked by loxP sites between a general promoter (CMV) and the mCherry coding sequence. Without Cre activity both GFP and the shRNA are expressed. Upon Cre-mediated recombination, the shRNA and GFP are excised, turning off their expression. Similar to (a), mCherry expression is activated following Cre-mediated recombination. (**c**) The Cre-loxP system can be used to label sparse cells. Cells are electroporated with three constructs: a fluorescent protein (GFP) containing a stop cassette flanked by loxP sites between a general promoter (CMV) and the GFP coding sequence, an RNAi construct, and a Cre recombinase under the control of a general or cell-specific promoter. Titration of the amount of the Cre construct results in partial expression, where a small fraction of the cells expresses Cre. While all cells are expressing the shRNA, only the cells with a Cre recombinase construct express GFP. (**d**) Implementation of the Cre-loxP system in the developing cortex labels sparse cells and allows detailed analysis of their morphology. Embryos were electroporated at E12.5 and harvested at E13.5 (our unpublished data). A Cre-loxP system as described at (c) was used to sparsely label post-mitotic neurons. The Cre recombinase was under the control of Tubulin α promoter.

Another technique to manipulate the population being knocked-down is the use of Cre-loxP mediated recombination [74,75,76]. The technique utilizes the Cre recombinase, which catalyzes the site-specific recombination events between two loxP sites. Several approaches use the Cre-mediated recombination technique (reviewed by [77]). In the conditional RNAi activation approach, Cre-mediated recombination excises an inhibitory sequence and activates the shRNA expression. To this end, the shRNA is separated from its promoter by a loxP-flunked stuffer sequence (Figure 2a). By the use of a Cre recombinase under the control of a cell-specific promoter, the shRNA expression is restricted to the cell population in which Cre recombinase will be expressed (see Table 3 and [65] for a review of cell-specific promoters used in the *in utero* electroporation system). Alternatively, in the conditional RNAi inactivation approach, the expression of the RNAi-construct can be expressed in all electroporated cells and specifically inactivated in a particular cell population. Here, the loxP sites flank the shRNA and its promoter and following Cre activity the shRNA will be lost (Figure 2b). Both approaches, conditional activation and inactivation, will usually require an additional fluorescent reporter which will mark the Cre expressing cells. Knockdown experiments usually allow easy visualization of the treated cells, which are coexpressing a fluorescent protein. Some analyses, for example, detailed neuronal morphology, require the labeling of sparse cells. Labeling of sparse cells can be achieved using *in utero* infection with low-titer of lentivirus, however, in case of *in utero* electroporation, usually multiple cells are transfected. To overcome this drawback, the expression of a fluorescent marker to label the cells can be titrated either by the amount of the plasmid introduced and/or the activity of Cre-loxP mediated recombination. In this case many of the cells in the electroporated area will express the shRNA; however, only a fraction of them will receive the Cre construct and will be GFP positive (Figure 2c,d). If a Cre-dependent shRNA is used with the Cre-dependent fluorescent marker and different concentrations of Cre recombinase, then only the labeled cells will express the GFP and the shRNA.

**Table 3:** Common methods to target different cortical populations.

***In utero* electroporation**	
*Electroporated area*	*Labeled population*
Ventricular zone of the dorsal telencephalon	Cortical progenitors and projection neurons
Lateral telencephalon/corticostriatal junction	Neurons of the amygdala and piriform cortex
Ganglionic eminences of the ventral telencephalon	Cortical interneurons
Hippocampal neuroepithelium	Hippocampal neurons
**Cre-Lox system**	
*Promoter*	*Labeled population*
BLBP, GFAP or GLAST	Radial glia [78]
Tubulin α	Post-mitotic neurons and neuronal progenitors [78]
Nestin	All ventricular zone progenitors [79]

Furthermore, it is possible to regulate the timing of expression by using a Cre-recombinase, which is activated following the addition of 4-hydroxytamoxifen. The Cre recombinase has been fused to a mutated ligand-binding domain of the human estrogen receptor (ER) resulting in a tamoxifen-dependent Cre recombinase, Cre-ERT, which is activated by tamoxifen, but not by estradiol [80]. The Cre-ERT system is currently being used mainly in transgenic mouse experiments [81], but is very suitable for *in utero* electroporation [65]. Another system for temporal regulation of the RNAi expression is the drug-inducible RNAi. The logic behind this system relies on drug-mediated control of the shRNA promoter, where the addition of the drug (e.g., doxycycline) sequesters a repressor of the promoter and allows the shRNA to be expressed [82,83,84]. The fundamental difference between this system and the Cre-inducible RNAi is the reversibility. That is, Cre-inducible RNAi is a one-time excision that cannot be restored to its original state. In drug-inducible RNAi, whilst the drug is delivered, the RNAi is expressed, whereas its removal restores the system to its original state. Finally, it is possible to combine *in utero* electroporation experiments with different transgenic mice. The combination of *in utero* electroporation gene transfer with the growing set of conditional RNAi systems and regulated Cre expression constructs provides a toolbox to precisely control the RNAi expression at almost any time in development and in any neuronal population.

### 2.6. Cell-Autonomous *versus* Non Cell-Autonomous Features

In transgenic mice cell autonomous features can be visualized using mosaic analysis with double markers (MADM) [85,86]. This system involves Cre-loxP-dependent recombination, however, the recombination is interchromosomal and mitosis dependent. The system involves formation of chimeric gene constructed from the *N*-terminal part of GFP together with the C-terminal part of DsRed (or another fluorescent protein) coding sequences. The parts of the fluorescent proteins are separated by an intron containing a loxP site, which are targeted to a chromosome containing the gene of interest to be mutated. The reciprocal chimeric gene, with the *N*-terminal part of DsRed and the *C*-terminal of GFP separated by an intron with a loxP site, is targeted to the reciprocal site of the WT chromosome. The resultant mice are then crossed to each other to generate the trans heterozygote mice. Cre-mediated recombination between the chromosomes results in green knockout cells, red WT cells and yellow heterozygous cells, the rest of the cells are colorless and heterozygous. Analyses of sparse and uniquely labeled mutant cells in mosaic animals reveal distinct cell-autonomous functions for the genes investigated. The system allows color-coding of the various genotypes and is very informative, nevertheless several drawbacks can be noted in comparison with the possible *in utero* electroporation methods. The MADM system is time-consuming and requires the generation of several knockin and knockout mice combined with a series of sequential mating of the respective mice. In addition, it should be noted that the background cells are heterozygous thus, may result in a phenotype different from the WT. 

It is possible to visualize cell-autonomous and non-cell autonomous features using *in utero* electroporation. One possibility is to use sequential *in utero* electroporation, where initially the shRNA transfected cells are labeled with GFP and then, following a time delay, cells are electroporated with a red fluorescent protein. This scenario was tested first in the case of *Dcx* shRNA, where the time interval between consecutive electroporations was thirty minutes [4]. In this sequential electroporation the migration of the green cells, which received the shRNA of *Dcx,* was inhibited in a cell-autonomous manner. However, the position of the red cells, neighboring the green cells, was also disrupted, thus demonstrating a non cell-autonomous effect. An alternative possibility is using dual colors, which will indicate the cells in which the shRNA is active or inactive following Cre-dependent recombination (Figure 2). For example, in Figure 2b, the cells expressing shRNA are also green, but following the activity of Cre recombinase the red fluorescent marker will be expressed and the shRNA with the green fluorescent protein will be deleted. The relative concentration of Cre recombinase can result in different fractions of labeled cells, which reside very close to each other. Thus, it may be possible to study the cell autonomous features of the green, treated cells and their effect on the red, control neighboring cells.

## 3. Conclusions and Perspectives

The use of shRNA to knockdown gene expression in the developing brain has provided a novel approach to understanding gene function, which differs from the knockout approach. This technology is amenable for moderate scale studies, thus it is possible to evaluate the role of several genes that are part of a gene family, or different members of a pathway. Conventional transgenic approaches will require an extended period of time to reach the desired double or triple mutant mice. Abnormal neuronal migration has been associated with many human diseases such as brain malformation, mental retardation, autism and schizophrenia (review [87]). Studies in the recent years indicate the presence of multiple candidate genes for schizophrenia, autism and mental retardation, through analysis of copy number variations or the presence of rare mutations [88,89,90,91,92,93,94,95,96,97,98,99]. Thus, there is an urgent necessity not only to understand the function of many genes during cortical development, but also to understand the role of single base changes, which result in pathogenic consequences. The possibility to reintroduce mutant genes on the basis of silenced genes has so far not been heavily utilized. We caution that shRNA-based experiments need to be tightly controlled to answer the specificity and efficacy requirements. However, in this field we are also viewing additional developments and advancements, wherein the future commercial companies will probably be preparing whole genome shRNA libraries verified for their ability to knockdown gene expression without affecting other non-target genes.

In addition, we foresee a possible increase in combining both *in utero* electroporation of shRNA with different lines of transgenic mice. There is a dramatic increase in the availability of different Cre lines of mice, which can be easily combined with conditional expression of the shRNA, as well as with conditional knockout. We believe that full understanding of gene function in the intricate processes involved in neuronal migration and brain structure formation will require the combination of multiple innovative techniques.

## References

[1] Dorsett Y., Tuschl T. (2004). SiRNAs: Applications in functional genomics and potential as therapeutics. Nature reviews. Drug Discov..

[2] Montgomery M.K., Xu S., Fire A. (1998). RNA as a target of double-stranded RNA-mediated genetic interference in caenorhabditis elegans. Proc. Natl. Acad. Sci. USA.

[3] Fire A., Xu S., Montgomery M.K., Kostas S.A., Driver S.E., Mello C.C. (1998). Potent and specific genetic interference by double-stranded RNA in caenorhabditis elegans. Nature.

[4] Bai J., Ramos R.L., Ackman J.B., Thomas A.M., Lee R.V., LoTurco J.J. (2003). RNAi reveals doublecortin is required for radial migration in rat neocortex. Nat. Neurosci..

[5] Mittal V. (2004). Improving the efficiency of RNA interference in mammals. Nat. Rev. Genet..

[6] Elbashir S.M., Harborth J., Lendeckel W., Yalcin A., Weber K., Tuschl T. (2001). Duplexes of 21-nucleotide RNAs mediate RNA interference in cultured mammalian cells. Nature.

[7] Bernstein E., Caudy A.A., Hammond S.M., Hannon G.J. (2001). Role for a bidentate ribonuclease in the initiation step of RNA interference. Nature.

[8] Zeng L., Gu S., Li Y., Zhao E., Xu J., Ye X., Wu Q., Wang L., Xie Y., Mao Y. (2003). Identification of a novel human doublecortin-domain-containing gene (DCDC1) expressed mainly in testis. J. Hum. Genet..

[9] Carmell M.A., Hannon G.J. (2004). RNase III enzymes and the initiation of gene silencing. Nat. Struct. Mol. Biol..

[10] Nykanen A., Haley B., Zamore P.D. (2001). ATP requirements and small interfering RNA structure in the RNA interference pathway. Cell.

[11] Martinez J., Patkaniowska A., Urlaub H., Luhrmann R., Tuschl T. (2002). Single-stranded antisense siRNAs guide target RNA cleavage in RANi. Cell.

[12] Khvorova A., Reynolds A., Jayasena S.D. (2003). Functional siRNAs and miRNAs exhibit strand bias. Cell.

[13] Schwarz D.S., Hutvagner G., Du T., Xu Z., Aronin N., Zamore P.D. (2003). Asymmetry in the assembly of the RNAi enzyme complex. Cell.

[14] Taxman D.J., Livingstone L.R., Zhang J., Conti B.J., Iocca H.A., Williams K.L., Lich J.D., Ting J.P., Reed W. (2006). Criteria for effective design, construction, and gene knockdown by shRAN vector. BMC Biotechnol..

[15] Reynolds A., Leake D., Boese Q., Scaringe S., Marshall W.S., Khvorova A. (2004). Rational siRAN design for RNA interference. Nat. Biotechnol..

[16] Yu J.Y., DeRuiter S.L., Turner D.L. (2002). RNA interference by expression of short-interfering RNAs and hairpin RNAs in mammalian cells. Proc. Natl. Acad. Sci. USA.

[17] Krichevsky A.M., Kosik K.S. (2002). RNAi functions in cultured mammalian neurons. Proc. Natl. Acad. Sci. USA.

[18] Kim D.H., Rossi J.J. (2007). Strategies for silencing human disease using RNA interference. Nat. Rev. Genet..

[19] Coquelle F.M., Levy T., Bergmann S., Wolf S.G., Bar-El D., Sapir T., Brody Y., Orr I., Barkai N., Eichele G., Reiner O. (2006). Common and divergent roles for members of the mouse dcx superfamily. Cell Cycle.

[20] Reiner O., Coquelle F.M., Peter B., Levy T., Kaplan A., Sapir T., Orr I., Barkai N., Eichele G., Bergmann S. (2006). The evolving doublecortin (dcx) superfamily. BMC Genomics.

[21] des Portes V., Pinard J.M., Billuart P., Vinet M.C., Koulakoff A., Carrie A., Gelot A., Dupuis E., Motte J., Berwald-Netter Y. (1998). A novel cns gene required for neuronal migration and involved in x-linked subcortical laminar hetrotropia and lissencephaly syndrome. Cell.

[22] Gleeson J.G., Allen K.M., Fox J.W., Lamperti E.D., Berkovic S., Scheffer I., Cooper E.C., Dobyns W.B., Minnerath S.R., Ross M.E. (1998). *Doublecortin*, a brain-specific gene mutated in human x-linked lissencephaly and double cortex syndrome, encodes a putative signaling protein. Cell.

[23] Le Hellard S., Havik B., Espeseth T., Breilid H., Lovlie R., Luciano M., Gow A.J., Harris S.E., Starr J.M., Wibrand K. (2009). Variants in doublecortin- and calmodulin kinase like 1, a gene up-regulated by BDNF, are associated with memory and general cognitive abilities. PLoS One.

[24] Lionel A.C., Crosbie J., Barbosa N., Goodale T., Thiruvahindrapuram B., Rickaby J., Gazzellone M., Carson A.R., Howe J.L., Wang Z. (2011). Rare copy number variation discovery and cross-disorder comparisons identify risk genes for ADHD. Sci. Transl. Med..

[25] Schumacher J., Anthoni H., Dahdouh F., Konig I.R., Hillmer A.M., Kluck N., Manthey M., Plume E., Warnke A., Remschmidt H. (2006). Strong genetic evidence of DCDC2 as a susceptibility gene for dyslexia. Am. J. Hum. Genet..

[26] Meng H., Smith S.D., Hager K., Held M., Liu J., Olson R.K., Pennington B.F., Defries J.C., Gelernter J., O’Reilly-Pol T. (2005). DCDC2 is associated with reading disability and modulates neuronal development in the brain. Proc. Natl. Acad. Sci. USA.

[27] Sullivan L.S., Heckenlively J.R., Bowne S.J., Zuo J., Hide W.A., Gal A., Denton M., Inglehearn C.F., Blanton S.H., Daiger S.P. (1999). Mutations in a novel retina-specific gene cause autosomal dominant retinitis pigmentosa. Nat. Genet..

[28] Conte I., Lestingi M., den Hollander A., Alfano G., Ziviello C., Pugliese M., Circolo D., Caccioppoli C., Ciccodicola A., Banfi S. (2003). Identification and characterisation of the retinitis pigmentosa 1-like1 gene (rp1l1): A novel candidate for retinal degenerations. Eur. J. Hum. Genet..

[29] Francis F., Koulakoff A., Boucher D., Chafey P., Schaar B., Vinet M.C., Friocourt G., McDonnell N., Reiner O., Kahn A. (1999). Doublecortin is a developmentally regulated, microtubule-associated protein expressed in migrating and differentiating neurons. Neuron.

[30] Nacher J., Crespo C., McEwen B.S. (2001). Doublecortin expression in the adult rat telencephalon. Eur. J. Neurosci..

[31] Saaltink D.J., Havik B., Verissimo C.S., Lucassen P.J., Vreugdenhil E. (2012). Doublecortin and doublecortin-like are expressed in overlapping and non-overlapping neuronal cell population: Implications for neurogenesis. J. Comp. Neurol..

[32] Friocourt G., Liu J.S., Antypa M., Rakic S., Walsh C.A., Parnavelas J.G. (2007). Both doublecortin and doublecortin-like kinase play a role in cortical interneuron migration. J. Neurosci..

[33] Bai J., Ramos R.L., Paramasivam M., Siddiqi F., Ackman J.B., LoTurco J.J. (2008). The role of dcx and lis1 in migration through the lateral cortical stream of developing forebrain. Dev. Neurosci..

[34] Ocbina P.J., Dizon M.L., Shin L., Szele F.G. (2006). Doublecortin is necessary for the migration of adult subventricular zone cells from neurospheres. Mol. Cell. Neurosci..

[35] Cohen D., Segal M., Reiner O. (2008). Doublecortin supports the development of dendritic arbors in primary hippocampal neurons. Dev. Neurosci..

[36] Shmueli O., Gdalyahu A., Sorokina K., Nevo E., Avivi A., Reiner O. (2001). DCX in PC12 cells: CREB-mediated transcription and neurite outgrowth. Hum. Mol. Genet..

[37] Burgess H.A., Martinez S., Reiner O. (1999). Kiaa0369, doublecortin-like kinase, is expressed during brain development. J. Neurosci. Res..

[38] Shu T., Tseng H.C., Sapir T., Stern P., Zhou Y., Sanada K., Fischer A., Coquelle F.M., Reiner O., Tsai L.H. (2006). Doublecortin-like kinase controls neurogenesis by regulating mitotic spindles and m phase progression. Neuron.

[39] Deuel T.A., Liu J.S., Corbo J.C., Yoo S.Y., Rorke-Adams L.B., Walsh C.A. (2006). Genetic interactions between doublecortin and doublecortin-like kinase in neuronal migration and axon outgrowth. Neuron.

[40] Verissimo C.S., Cheng S., Puigvert J.C., Qin Y., Vroon A., van Deutekom J., Price L.S., Danen E.H., van de Water B., Fitzsimons C.P. (2012). Combining doublecortin-like kinase silencing and vinca alkaloids results in a synergistic apoptotic effect in neuroblastoma cells. J. Pharmacol. Exp. Ther..

[41] Verissimo C.S., Molenaar J.J., Meerman J., Puigvert J.C., Lamers F., Koster J., Danen E.H., van de Water B., Versteeg R., Fitzsimons C.P. (2010). Silencing of the microtubule-associated proteins doublecortin-like and doublecortin-like kinase-long induces apoptosis in neuroblastoma cells. Endoc. Relat. Cancer.

[42] Schenk G.J., Vreugdenhil E., Hubens C.J., Veldhuisen B., de Kloet E.R., Oitzl M.S. (2011). Hippocampal carp over-expression solidifies consolidation of contextual fear memories. Physiol. Behav..

[43] Schenk G.J., Werkman T., Wadman W., Veldhuisen B., Dijkmans T.F., Blaas E., Kegel L., de Kloet E.R., Vreugdenhil E. (2010). Over-expression of the dclk gene transcript carp decreases CA3/CA1 network excitability. Brain Res..

[44] Schenk G.J., Veldhuisen B., Wedemeier O., McGown C.C., Schouten T.G., Oitzl M., de Kloet E.R., Vreugdenhil E. (2010). Over-expression of deltac-dclk-short in mouse brain results in a more anxious behavioral phenotype. Physiol. Behav..

[45] Edelman A.M., Kim W.Y., Higgins D., Goldstein E.G., Oberdoerster M., Sigurdson W. (2005). Doublecortin kinase-2, a novel doublecortin-related protein kinase associated with terminal segments of axons and dendrites. J. Biol. Chem..

[46] Tuy F.P., Saillour Y., Kappeler C., Chelly J., Francis F. (2008). Alternative transcripts of Dclk1 and Dclk2 and their expression in doublecortin knockout mice. Dev. Neurosci..

[47] Kerjan G., Koizumi H., Han E.B., Dube C.M., Djakovic S.N., Patrick G.N., Baram T.Z., Heinemann S.F., Gleeson J.G. (2009). Mice lacking doublecortin and doublecortin-like kinase 2 display altered hippocampal neuronal maturation and spontaneous seizures. Proc. Natl. Acad. Sci. USA.

[48] Ohmae S., Takemoto-Kimura S., Okamura M., Adachi-Morishima A., Nonaka M., Fuse T., Kida S., Tanji M., Furuyashiki T., Arakawa Y. (2006). Molecular identification and characterization of a family of kinases with homology to camki/camkiv. J. Biol. Chem..

[49] Liu Q., Zuo J., Pierce E.A. (2004). The retinitis pigmentosa 1 protein is a photoreceptor microtubule-associated protein. J. Neurosci..

[50] Gao J., Cheon K., Nusinowitz S., Liu Q., Bei D., Atkins K., Azimi A., Daiger S.P., Farber D.B., Heckenlively J.R. (2002). Progressive photoreceptor degeneration, outer segment dysplasia, and rhodopsin mislocalization in mice with targeted disruption of the retinitis pigmentosa-1 (Rp1) gene. Proc. Natl. Acad. Sci. USA.

[51] Massinen S., Hokkanen M.E., Matsson H., Tammimies K., Tapia-Paez I., Dahlstrom-Heuser V., Kuja-Panula J., Burghoorn J., Jeppsson K.E., Swoboda P. (2011). Increased expression of the dyslexia candidate gene DCDC2 affects length and signaling of primary cilia in neurons. PLoS One.

[52] Kaplan A., Reiner O. (2011). Linking cytoplasmic dynein and transport of rab8 vesicles to the midbody during cytokinesis by the doublecortin domain-containing 5 protein. J. Cell. Sci..

[53] Corbo J.C., Deuel T.A., Long J.M., LaPorte P., Tsai E., Wynshaw-Boris A., Walsh C.A. (2002). Doublecortin is required in mice for lamination of the hippocampus but not the neocortex. J. Neurosci..

[54] Ramos R.L., Bai J., LoTurco J.J. (2006). Heterotopia formation in rat but not mouse neocortex after RNA interference knockdown of dcx. Cereb. Cortex.

[55] Koizumi H., Tanaka T., Gleeson J.G. (2006). Doublecortin-like kinase functions with doublecortin to mediate fiber tract decussation and neuronal migration. Neuron.

[56] Koizumi H., Higginbotham H., Poon T., Tanaka T., Brinkman B.C., Gleeson J.G. (2006). Doublecortin maintains bipolar shape and nuclear translocation during migration in the adult forebrain. Nat. Neurosci..

[57] Wang Y., Yin X., Rosen G., Gabel L., Guadiana S.M., Sarkisian M.R., Galaburda A.M., Loturco J.J. (2011). DCDC2 knockout mice display exacerbated developmental disruptions following knockdown of doublecortin. Neuroscience.

[58] Jackson A.L., Linsley P.S. (2010). Recognizing and avoiding siRNA off-target effects for target identification and therapeutic application. Nat. Rev. Drug Discov..

[59] Cullen B.R. (2006). Enhancing and confirming the specificity of RNAi experiments. Nat. Methods.

[60] Jackson A.L., Bartz S.R., Schelter J., Kobayashi S.V., Burchard J., Mao M., Li B., Cavet G., Linsley P.S. (2003). Expression profiling reveals off-target gene regulation by RNAi. Nat. Biotechnol..

[61] Hüttenhofer A., Schattner P., Hall J., Mattick J.S., Brummelkamp T.R., Bernards R., Martienssen R.A. (2003). Whither RNAi?. Nat. Cell. Biol..

[62] Sapir T., Sapoznik S., Levy T., Finkelshtein D., Shmueli A., Timm T., Mandelkow E.M., Reiner O. (2008). Accurate balance of the polarity kinase MARK2/Par-1 is required for proper cortical neuronal migration. J. Neurosci..

[63] LoTurco J.J., Bai J. (2006). The multipolar stage and disruptions in neuronal migration. Trends Neurosci..

[64] Manent J.B., Wang Y., Chang Y., Paramasivam M., LoTurco J.J. (2009). Dcx reexpression reduces subcortical band heterotopia and seizure threshold in an animal model of neuronal migration disorder. Nat. Med..

[65] LoTurco J., Manent J.B., Sidiqi F. (2009). New and improved tools for in utero electroporation studies of developing cerebral cortex. Cereb. Cortex.

[66] Schaar B.T., Kinoshita K., McConnell S.K. (2004). Doublecortin microtubule affinity is regulated by a balance of kinase and phosphatase activity at the leading edge of migrating neurons. Neuron.

[67] Sapir T., Shmueli A., Levy T., Timm T., Elbaum M., Mandelkow E.M., Reiner O. (2008). Antagonistic effects of doublecortin and MARK2/Par-1 in the developing cerebral cortex. J. Neurosci..

[68] Tabata H., Nakajima K. (2001). Efficient in utero gene transfer system to the developing mouse brain using electroporation: Visualization of neuronal migration in the developing cortex. Neuroscience.

[69] Saito T., Nakatsuji N. (2001). Efficient gene transfer into the embryonic mouse brain using *in vivo* electroporation. Dev. Biol..

[70] Borrell V., Yoshimura Y., Callaway E.M. (2005). Targeted gene delivery to telencephalic inhibitory neurons by directional *in utero* electroporation. J. Neurosci. Methods.

[71] Navarro-Quiroga I., Chittajallu R., Gallo V., Haydar T.F. (2007). Long-term, selective gene expression in developing and adult hippocampal pyramidal neurons using focal *in utero* electroporation. J. Neurosci..

[72] Angevine J.B., Sidman R.L. (1961). Autoradiographic study of cell migration during histogenesis of cerebral cortex in the mouse. Nature.

[73] Langevin L.M., Mattar P., Scardigli R., Roussigne M., Logan C., Blader P., Schuurmans C. (2007). Validating *in utero* electroporation for the rapid analysis of gene regulatory elements in the murine telencephalon. Dev. Dyn..

[74] Matsuda T., Cepko C.L. (2007). Controlled expression of transgenes introduced by *in vivo* electroporation. Proc. Natl. Acad. Sci. USA.

[75] Konno D., Shioi G., Shitamukai A., Mori A., Kiyonari H., Miyata T., Matsuzaki F. (2008). Neuroepithelial progenitors undergo lgn-dependent planar divisions to maintain self-renewability during mammalian neurogenesis. Nat. Cell Biol..

[76] Morin X., Jaouen F., Durbec P. (2007). Control of planar divisions by the g-protein regulator lgn maintains progenitors in the chick neuroepithelium. Nat. Neurosci..

[77] Wiznerowicz M., Szulc J., Trono D. (2006). Tuning silence: Conditional systems for RNA interference. Nat. Methods.

[78] Gal J.S., Morozov Y.M., Ayoub A.E., Chatterjee M., Rakic P., Haydar T.F. (2006). Molecular and morphological heterogeneity of neural precursors in the mouse neocortical proliferative zones. J. Neurosci..

[79] Malatesta P., Hack M.A., Hartfuss E., Kettenmann H., Klinkert W., Kirchhoff F., Gotz M. (2003). Neuronal or glial progeny: Regional differences in radial glia fate. Neuron.

[80] Feil R., Brocard J., Mascrez B., LeMeur M., Metzger D., Chambon P. (1996). Ligand-activated site-specific recombination in mice. Proc. Natl. Acad. Sci. USA.

[81] Hayashi S., McMahon A.P. (2002). Efficient recombination in diverse tissues by a tamoxifen-inducible form of cre: A tool for temporally regulated gene activation/inactivation in the mouse. Dev. Biol..

[82] Stegmeier F., Hu G., Rickles R.J., Hannon G.J., Elledge S.J. (2005). A lentiviral microRNA-based system for single-copy polymerase ii-regulated RNA interference in mammalian cells. Proc. Natl. Acad. Sci. USA.

[83] Wiznerowicz M., Trono D. (2003). Conditional suppression of cellular genes: Lentivirus vector-mediated drug-inducible RNA interference. J. Virol..

[84] Ohkawa J., Taira K. (2000). Control of the functional activity of an antisense RNA by a tetracycline-responsive derivative of the human u6 snRNA promoter. Hum. Gene Ther..

[85] Hippenmeyer S., Youn Y.H., Moon H.M., Miyamichi K., Zong H., Wynshaw-Boris A., Luo L. (2010). Genetic mosaic dissection of lis1 and ndel1 in neuronal migration. Neuron.

[86] Zong H., Espinosa J.S., Su H.H., Muzumdar M.D., Luo L. (2005). Mosaic analysis with double markers in mice. Cell.

[87] Reiner O., Sapoznik S., Sapir T. (2006). Lissencephaly 1 linking to multiple diseases: Mental retardation, neurodegeneration, schizophrenia, male sterility, and more. Neuromol. Med..

[88] Sanders S.J., Murtha M.T., Gupta A.R., Murdoch J.D., Raubeson M.J., Willsey A.J., Ercan-Sencicek A.G., DiLullo N.M., Parikshak N.N., Stein J.L. (2012). *De novo* mutations revealed by whole-exome sequencing are strongly associated with autism. Nature.

[89] O'Roak B.J., Vives L., Girirajan S., Karakoc E., Krumm N., Coe B.P., Levy R., Ko A., Lee C., Smith J.D. (2012). Sporadic autism exomes reveal a highly interconnected protein network of de novo mutations. Nature.

[90] Neale B.M., Kou Y., Liu L., Ma’ayan A., Samocha K.E., Sabo A., Lin C.F., Stevens C., Wang L.S., Makarov V. (2012). Patterns and rates of exonic *de novo* mutations in autism spectrum disorders. Nature.

[91] Levy D., Ronemus M., Yamrom B., Lee Y.H., Leotta A., Kendall J., Marks S., Lakshmi B., Pai D., Ye K. (2011). Rare *de novo* and transmitted copy-number variation in autistic spectrum disorders. Neuron.

[92] Gilman S.R., Iossifov I., Levy D., Ronemus M., Wigler M., Vitkup D. (2011). Rare *de novo* variants associated with autism implicate a large functional network of genes involved in formation and function of synapses. Neuron.

[93] Cooper G.M., Coe B.P., Girirajan S., Rosenfeld J.A., Vu T.H., Baker C., Williams C., Stalker H., Hamid R., Hannig V. (2011). A copy number variation morbidity map of developmental delay. Nat. Genet..

[94] Kirov G., Grozeva D., Norton N., Ivanov D., Mantripragada K.K., Holmans P., Craddock N., Owen M.J., O’Donovan M.C. (2009). Support for the involvement of large copy number variants in the pathogenesis of schizophrenia. Hum. Mol. Genet..

[95] Elia J., Gai X., Xie H.M., Perin J.C., Geiger E., Glessner J.T., D’Arcy M., deBerardinis R., Frackelton E., Kim C. (2010). Rare structural variants found in attention-deficit hyperactivity disorder are preferentially associated with neurodevelopmental genes. Mol. Psychiatry.

[96] Xu B., Roos J.L., Levy S., van Rensburg E.J., Gogos J.A., Karayiorgou M. (2008). Strong association of de novo copy number mutations with sporadic schizophrenia. Nat. Genet..

[97] Walsh T., McClellan J.M., McCarthy S.E., Addington A.M., Pierce S.B., Cooper G.M., Nord A.S., Kusenda M., Malhotra D., Bhandari A. (2008). Rare structural variants disrupt multiple genes in neurodevelopmental pathways in schizophrenia. Science.

[98] Stone J.L., O’Donovan M.C., Gurling H., Kirov G.K., Blackwood D.H., Corvin A., Craddock N.J., Gill M., Hultman C.M., Lichtenstein P. (2008). Rare chromosomal deletions and duplications increase risk of schizophrenia. Nature.

[99] Stefansson H., Rujescu D., Cichon S., Pietilainen O.P., Ingason A., Steinberg S., Fossdal R., Sigurdsson E., Sigmundsson T., Buizer-Voskamp J.E. (2008). Large recurrent microdeletions associated with schizophrenia. Nature.

